# Bioactive Compounds, Health Benefits and Food Applications of Grape

**DOI:** 10.3390/foods11182755

**Published:** 2022-09-07

**Authors:** Dan-Dan Zhou, Jiahui Li, Ruo-Gu Xiong, Adila Saimaiti, Si-Yu Huang, Si-Xia Wu, Zhi-Jun Yang, Ao Shang, Cai-Ning Zhao, Ren-You Gan, Hua-Bin Li

**Affiliations:** 1 Guangdong Provincial Key Laboratory of Food, Nutrition and Health, Department of Nutrition, School of Public Health, Sun Yat-sen University, Guangzhou 510080, China; 2 School of Science, The Hong Kong University of Science and Technology, Hong Kong 999077, China; 3 School of Chinese Medicine, Li Ka Shing Faculty of Medicine, The University of Hong Kong, Hong Kong 999077, China; 4 Department of Clinical Oncology, Li Ka Shing Faculty of Medicine, The University of Hong Kong, Hong Kong 999077, China; 5 Research Center for Plants and Human Health, Institute of Urban Agriculture, Chinese Academy of Agricultural Sciences, Chengdu 610213, China

**Keywords:** grape, grape seed, bioactive compounds, antioxidant, health benefits, application

## Abstract

Grape (*Vitis vinifera* L.) is one of the most popular fruits worldwide. It contains various bioactive compounds, such as proanthocyanidins, anthocyanins, flavonols, phenolic acids and stilbenes, the contents of which could vary considerably in grape skin, pulp and seed. Many studies have revealed that grape possesses a variety of health benefits, such as antioxidant, anti-inflammatory, gut-microbiota-modulating, anticancer and cardioprotective effects. Grape is eaten as fresh fruit and is also used as raw material to produce various products, such as wine, grape juice and raisins. Moreover, the byproducts of grape, such as grape pomace and grape seed, have many applications in the food industry. In this paper, the bioactive compounds in grape are briefly summarized based on literature published in recent years. In addition, the health benefits of grape and its bioactive components are discussed, with special attention paid to the underlying mechanisms. Furthermore, the applications of grape in the food industry are elucidated, especially the applications of grape pomace and grape seed. This paper can contribute to understanding the health benefits and mechanisms of grape and its bioactive compounds, as well as the promotion of the use of grape in the food industry.

## 1. Introduction

Grape (*Vitis vinifera* L.) is a delicious and nutritious fruit with a long cultivation history and is still among the most produced fruits in the world [1]. The annual production of grape is approximately 75 million tons, with the largest production in Europe (about 41%), followed by Asia (29%) and the Americas (21%) [2,3]. Many studies have reported that grape exhibits a variety of biological activities, such as antioxidant [4,5], gut-microbiota-regulating [6], cardioprotective [7,8], antidiabetic [9] and anticancer activities [10,11]. For example, a double-blind, placebo-controlled randomized clinical trial (RCT) revealed that the consumption of 46 g lyophilized grape powder (equivalent to 252 g fresh grape) resulted in antioxidant and anti-inflammatory activities in men with metabolic syndrome in the absence of dyslipidemia [12]. Another RCT showed that daily intake of 100 mg grape seed extract for 8 weeks significantly improved insulin resistance in adolescents with metabolic syndrome [13]. The health benefits of grape are largely attributed to its rich bioactive compounds, especially polyphenols, which mainly consist of proanthocyanidins, anthocyanins, flavonols, phenolic acids and stilbenes [8,11]. Polyphenol contents can vary considerably depending on the fractions of grape. Grape seed contains the majority of polyphenols, mainly proanthocyanidins [14,15]. Grape skin contains relatively high amounts of anthocyanins, whereas almost no anthocyanins are found in grape seed [16,17].

Grape is eaten as fresh fruit, with a variety of applications in the food industry. Many grape-derived products are manufactured and sold, such as wine, grape juice, grape jam and raisins [18]. Winemaking is the most prominent application of grape, consuming approximately 50% of grape crops [3]. In addition, with the expansive production of grape products, byproducts (such as grape pomace and grape seed) are produced in a considerable amounts, which could cause many environmental issues and represent a waste of resources [19,20]. The utilization of these byproducts has attracted increasing attention. Many studies have shown that grape byproducts have considerable application potential in the food industry. In particular, some products derived from grape seed have been sold commercially, such as grape seed extract capsules and grape seed oil [21,22]. In this review, we collect related literature published in recent years from the Web of Science Core Collection and PubMed databased and discuss the major bioactive compounds in grape. Moreover, we summarize the health benefits of grape and elaborate the underlying mechanisms. In addition, we discuss the applications of grape in the food industry, with special attention paid to the applications of grape pomace and grape seed. This paper provides an up-to-date summary of research progress with respect to grape, contributing to improved understanding of the relationship between grape, as well as its main bioactive compounds, and human health, in addition to promoting the application of grape in the food industry.

## 2. Bioactive Compounds in Grape

**Figure 1:**
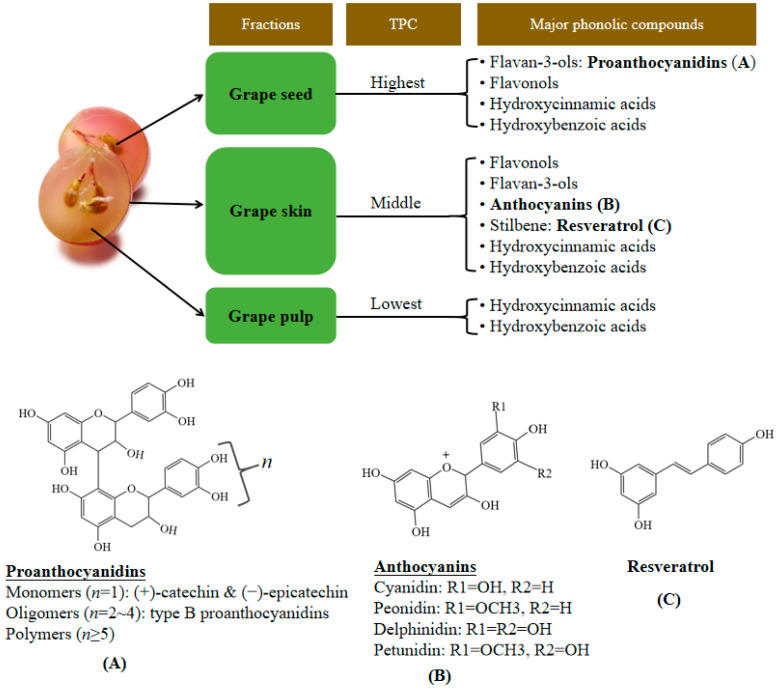
Phenolic compounds in different fractions of grape and the chemical structures of several representative bioactive compounds. (**A**) Proanthocyanidins; (**B**) anthocyanins; (**C**) resveratrol. TPC, total phenolic content.

Grape is an excellent source of bioactive compounds, especially polyphenols (Figure 1), which endows grape with a variety of biological activities [8,23]. Although polyphenols abound in grape, their contents differ considerably depending on the grape fraction. The highest total phenolic content (TPC) is found in grape seed, followed by grape skin and pulp [24,25]. The average TPC in grape seed is reported to be 130 fold higher that in grape pulp (mg gallic acid equivalent (GAE)/g dry weight) [24]. The composition of polyphenols in grape is complex. A total of 78 phenolic compounds were tentatively identified in grape pulp samples with LC-ESI-QTOF-MS/MS, as listed in Table 1 [26]. The major flavonoids in grape include flavan-3-ols (flavanols), flavonols and anthocyanins, whereas non-flavonoids mainly consist of phenolic acids and stilbenes. Flavan-3-ols are the richest flavonoids in grape and are mostly stored in the seeds but barely detected in the flesh [16]. Proanthocyanidins account for the majority of flavan-3-ols in grape, which are a class of compounds synthesized by the aggregation of flavanol monomers. Proanthocyanidins in grape include proanthocyanidin monomers, proanthocyanidin oligomers and more complicated proanthocyanidin polymers, among which (+)-catechin, (−)-epicatechin, procyanidin B1 and procyanidin B2 are the four major proanthocyanidins [14]. Flavonols accumulate considerably in grape skin and can also be found in the seeds of some grape varieties, including quercetin, myricetin and kaempferol [27,28]. Anthocyanins are water-soluble pigments that are mostly concentrated in the skin of colored grape cultivars (such as red or purple grapes), whereas anthocyanins are reported to be absent in white grape cultivars [29]. 

Resveratrol (3,5,4′-trihydroxystilbene) is the most important stilbene in grape, mainly presenting in the trans form. Grape skin is the major reservoir of resveratrol, which is a synthesized phytoalexin acting as a defense mechanism against pathogenic attack and environmental stress, such as fungal infection, injury and UV irradiation [11,30]. In addition, a variety of phenolic acids exist in grape in either free or conjugated forms, with hydroxybenzoic and hydroxycinnamic acids being the most abundant. Hydroxybenzoic acids in grape include 4-hydroxybenzoic, gallic, protocatechuic, vanillic and syringic acids, and major hydroxycinnamic acids include p-coumaric, caffeic, ferulic and sinapic acids [16,31,32]. 

Grape and grape-derived products also contain melatonin [33]. Melatonin, resveratrol and anthocyanins are three of the four most studied natural compounds, in addition to curcumin [34,35]. In this paper, we will focus on the health benefits of grape and its by-products but not single or pure resveratrol, anthocyanins and melatonin because their bioactivities have been widely reviewed in the literature in recent years [36,37,38].

## 3. Health Benefits of Grape

Grape exerts diverse health benefits, such as antioxidant, anti-inflammatory, gut-microbiota-modulating, antiobesity, cardioprotective, hepatoprotective, antidiabetic and anticancer effects, which will be discussed in detail below.

### 3.1. Antioxidant Activity

Oxidative stress is characterized by an imbalanced status between oxidation and antioxidation in the body and plays an important role in accelerating aging and inducing various metabolic diseases [39,40]. Grape has been reported to exhibit excellent in vitro and in vivo antioxidant ability. For example, a study showed that 13 Spanish red and white grape varieties exhibited in vitro antioxidant activity, including oxygen radical absorbance capacity (ORAC) (254.94 ± 12.06–412.90 ± 9.67 (for red grape) and 102.28 ± 2.22–440.11 ± 39.33 (for white grape) µmol Trolox equivalents (TE)/g dry weight (DW)), 2,2-diphenyl-1-picrylhydrazyl (DPPH), radical scavenging activity (72.95 ± 2.21–230.67 ± 11.71 (for red grape) and 29.50 ± 1.57–206.80 ± 10.45 (for white grape) µmol TE/g DW), ferric-reducing antioxidant capacity (FRAP) (54.43 ± 0.34–124.38 ± 2.06 (for red grape) and 14.36 ± 0.33–114.06 ± 10.44 (for white grape) µmol TE/g DW), cupric-reducing antioxidant capacity (CUPRAC) (96.24 ± 1.23–201.78 ± 8.91 (for red grape) and 45.54 ± 2.63–189.49 ± 8.21 (for white grape) µmol TE/g DW) and 2,2′-azino-bis (3-ethylbenzothiazoline-6-sulfonic acid) (ABTS) radical-scavenging activity (159.79 ± 7.10–220.82 ± 4.48 (for red grape) and 129.87 ± 13.49–288.75 ± 1.26 (for white grape) µmol TE/g DW) [41]. The antioxidant capacity of grape is strongly dependent on its TPC because the phenolic groups in the chemical structures of polyphenols are able to reduce reactive oxygen species (ROS) and chelate metal ions. In addition, different fractions of grape showed different antioxidant abilities. In our previous studies, the antioxidant activities of pulp, peel and seeds of 30 grape varieties were evaluated. The results showed that grape seeds possessed the strongest antioxidant activity, with the highest FRAP and Trolox equivalent antioxidant capacity (TEAC) values (FRAP: 312.429–858.121 µmol Fe(II)/g fresh weight (FW); TEAC: 207.815–473.454 µmol Trolox/g FW), followed by peel (FRAP: 18.304–252.983 µmol Fe(II)/g FW; TEAC: 5.176–123.740 µmol Trolox/g FW) and pulp (FRAP: 1.289–11.767 µmol Fe(II)/g FW; TEAC: 0.339–4.839 µmol Trolox/g FW), and these antioxidant values were in accordance with the respective TPC values because the majority of polyphenols were concentrated in the grape seeds, followed by peel and pulp [4,19]. 

Some cell and animal studies have explored the antioxidant capacity of grape and its underlying mechanisms. A study by Nallathambi et al. showed that grape seed extract significantly decreased the production of intracellular ROS and mitochondrial superoxide induced by lipopolysaccharide (LPS) in human Caco-2 colon cells and upregulated the gene expression of antioxidant enzymes, such as glutathione-disulfide reductase (GSR), superoxide dismutase (SOD) and glutathione peroxidase (GP_X_) [42]. In another study, grape peel powder ameliorated 2,4,6-trinitrobenzene sulfonic acid (TNBS)-induced oxidative damage in rats by restoring the activities of antioxidant enzymes, such as catalase (CAT) and SOD, and decreasing oxidative and NO levels in the colon [43]. Proanthocyanidins have been reported to play an important role in the antioxidant activity of grape. A cell study by He et al. showed that grape seed proanthocyanidins protected PC12 cells from oxidative injury induced by H_2_O_2_ via the phosphoinositide 3-kinase (PI3K)/serine-threonine kinase (Akt) signaling pathway [44]. In addition, grape seed proanthocyanidin extract ameliorated varicocele-induced testicular oxidative damage in rats by activating the nuclear factor (erythroid-derived 2)-like 2 (Nrf2) pathway [45].

Collectively, grape showed a strong antioxidant activity, which was mainly by increasing the activity of antioxidant enzymes and regulating the involved signaling pathways, such as PI3K/Akt and Nrf2 pathways. Different fractions of grape exhibit different antioxidant activities based on the content of polyphenols. Grape seed, the major reservoir of phenolic compounds, possesses the highest antioxidant ability, and proanthocyanidins are among the most important phenolic compounds contributing to the antioxidant activity of grape. 

### 3.2. Anti-Inflammatory Activity

Inflammation is closely associated with the development of pathological conditions, such as metabolic syndrome and several chronic diseases [46]. Grape exhibits considerable effectiveness in fighting inflammation. For instance, a study revealed that whole grape powder significantly inhibited human tumor necrosis factor (TNF)-mediated inflammation and alleviated the symptoms of inflammatory arthritis in transgenic mice overexpressing TNF [47]. Another study compared the anti-inflammatory activity of 16 varieties of table and wine grapes in gastric epithelial cells, revealing that two grape varieties (Exalta grape and Albarossa grape) showed the most active effect with respect to inhibiting TNF-α-induced interleukin (IL)-8 release because they were particularly rich in polyphenols [2]. This study further revealed that grape seed and skin portions with rich polyphenols were closely associated with anti-inflammatory effect. 

The anti-inflammatory mechanisms of grape, as well as its bioactive compounds, include inhibiting the secretion of proinflammatory cytokines and modulation of the related signaling pathways. A study revealed that TNBS-induced colitis in Wistar rats increased the levels of proinflammatory cytokines (such as TNF-α and IL-1β) in serum and colon tissue, whereas grape peel powder attenuated the inflammatory response by downregulating the nuclear factor kappa B (NF-κB) pathway [43]. In addition, diet supplementation with 8% grape seed meal counteracted dextran-sulfate-sodium-induced inflammation in the colon of pigs by inhibiting mitogen-activated protein kinase (MAPK) signaling cascades, downregulating NF-κB gene and protein expression and decreasing the production of proinflammatory cytokines (such as TNF-β, TNF-α and IL-6) and chemokines (such as IL-8 and macrophage inflammatory protein-1α) [48]. Polyphenols are the bioactive compounds in grape and contribute to its anti-inflammatory activity. It was reported that grape seed proanthocyanidins can inhibit inflammation in cigarette-smoke-exposed pulmonary arterial hypertension rats through the peroxisome-proliferator-activated receptor γ (PPAR-γ)/cyclooxygenase 2 (COX-2) pathway [49]. 

In brief, many studies have revealed the anti-inflammatory activity of grape, as well as its bioactive constituents, particularly polyphenols. The anti-inflammatory mechanisms of grape are mainly related to a decrease in the secretion of proinflammatory cytokines, such as TNF-α, IL-6 and IL-1β, and regulation of the related signaling pathways, such as PPAR-γ/COX-2, MAPK and NF-κB pathways.

### 3.3. Gut Microbiota Modulation

Gut microbiota is the large and complex community of microorganisms inhabiting the intestine that helps to build vital metabolic and immune functions, exerting a marked effect on the nutritional and health conditions of the host [50]. Dysregulation of the composition and diversity of gut microbiota is closely associated with metabolic syndrome and various diseases, such as allergies, diabetes, obesity and immune disorders [50]. In recent years, increasing attention has been focused on the efficacy of grape in modulating gut microbiota. It was reported that grape extract restored the dysbiosis of gut microbiota induced by a high-fat diet by increasing the *Firmicutes*/*Bacteroidetes* ratio and the abundances of *Bifidobacteria*, *Clostridia* and *Akkermansia* genera [6]. In addition, grape seed extract effectively maintained the homeostasis of gut microbiota and ameliorated 2-Amino-1-methyl-6-phenylimidazo [4,5-b] pyridine (PhIP)-induced colonic injury in Wistar rats, which was achieved by preventing a PhIP-mediated reduction in *Lactobacillus* abundance [51]. Antibiotics are widely used in clinical setting to treat infectious diseases, although they have been found to alter the gut microbiota and increase the risk of many metabolic diseases. A study revealed that grape seed polyphenol extract had a positive influence on the recovery of gut microbiota after treatment with an antibiotic cocktail in high-fat-diet-fed mice [52]. In brief, grape exhibited a potent ability to modulate the gut microbiota, and polyphenols were found to play an important role in this ability.

### 3.4. Antiobesity Activity

Obesity is a commonly occurring chronic metabolic disease worldwide that is characterized by excessive adipose accumulation and/or abnormal adipose distribution in the body and could be caused by multiple factors, such as a high-calorie diet, insufficient exercise and genetic inheritance [53]. Obesity is closely associated with many diseases, such as diabetes mellitus, cardiovascular disease and cancer, and has become a global public health concern [54]. Growing evidence have revealed the antiobesity activity of grape extract, and the relevant mechanisms have also been studied. Grape extract was reported to prevent obesity in high-fat- and high-fructose-diet-fed mice by restoring the dysbiosis of the gut microbiota. It regulated serum bile acid and promoted G-protein-coupled bile acid receptor 1 in brown adipose tissue, which activated thermogenesis of brown adipose tissue and increased energy consumption [6]. Another study revealed that grape seed flour ameliorated body weight gain and improved hepatic and serum lipid profiles in high-fat-diet-induced obese mice by promoting the thermogenesis of brown adipose tissue and increasing energy expenditure [55]. In addition, grape seed proanthocyanidin extract significantly reduced body weight, liver weight and fat weight of epididymal white adipose tissue, retroperitoneal white adipose tissue and inguinal white adipose tissue in high-fat-diet-fed C57BL/6 J mice by stimulating the thermogenic function of adipose tissue, promoting the browning of white adipose tissue and regulating the gut microbiota [56]. 

Sierra-Cruz et al. used a cafeteria diet to induce obesity and evaluated the antiobesity effect of grape seed proanthocyanidin extract on aged rats at two time points (a 10-day preventive treatment prior to cafeteria diet and a simultaneous treatment with the cafeteria diet for 11 weeks). The results showed that simultaneous treatment with the extract and cafeteria diet effectively reduced body weight, total adiposity and liver steatosis, whereas the preventive treatment only reduced mesenteric adiposity [57]. Human evidence is important to verify the antiobesity effect of grape. A double-blind RCT assigned 40 obese or overweight subjects to receive grape seed extract (300 mg/day) or placebo under a calorie-restricted diet for 12 weeks, and the results showed that the grape seed extract group had significantly lower body weight, body mass index, waist circumference and waist-to-hip ratio compared to the placebo group at the end of the study period [58]. However, human evidence is limited, and additional clinical studies are needed to further confirm the antiobesity effect of grape extract in the future.

In general, grape extract and its bioactive compounds exhibit excellent antiobesity potential, and the underlying mechanisms have been associated with the regulation of the gut microbiota, thermogenesis of adipose tissue and browning of white adipose tissue. Few clinical studies have been conducted to verify the antiobesity effect of grape extract in human beings; therefore, more of such studies should be conducted in the future.

### 3.5. Cardioprotective Activity

Cardiovascular disease is the leading cause of death worldwide [59]. Grape and its bioactive compounds have been reported to exert excellent efficacy in the prevention and treatment of cardiovascular disease. Hypertension is among the most prominent risk factors for cardiovascular disease [60]. Grape exhibits hypotensive activity, which could effectively prevent the development of cardiovascular diseases. For example, in vitro and in vivo experimental results showed that grape extract from chardonnay (a grape cultivar for white wine) increased nitric oxide (NO) production in cultured endothelial cells and ameliorated endothelial dysfunction and hypertension induced by deoxycorticosterone acetate-salt in rats by activating the endothelial NO synthase and PI3K/Akt pathways [61]. In addition, grape seed proanthocyanidin ameliorated left ventricular remodeling in spontaneously hypertensive rats by lowering SBP, reducing oxidative stress and regulating the levels of vasoactive substances [62]. 

Myocardial infarction is a severe and common type of cardiovascular disease caused by acute occlusion of coronary arteries. The results of a study by Svezia et al. suggest that pure grape juice protected the myocardium from an ischemic microenvironment and protected infarcted hearts by regulating gene expression of C-type natriuretic peptide, which is among the major autocrine/paracrine regulators of cardiac remodeling in infarcted hearts, acting as an endothelium-derived hyperpolarizing factor, modulating vascular tension and blood flow [7]. In addition, grape seed proanthocyanidin extract effectively improved cardiac remodeling and dysfunction caused by myocardial infarction in mice and protected cardiomyocytes from apoptosis in hypoxic situations through the PI3K/Akt pathway [63].

Many clinical studies have evaluated the cardioprotective activity of grape. A randomized, double-blind, crossover test revealed that acute grape seed extract supplementation significantly reduced SBP and mean arterial pressure (MAP) in both normal-body-weight males (SBP: 103 ± 4 vs. 99 ± 3 mmHg; MAP: 75 ± 2 vs. 72 ± 2 mmHg) and obese males (SBP: 118 ± 3 vs. 112 ± 5 mmHg; MAP: 86 ± 3 vs. 84 ± 3 mmHg), and the effect in obese males might be associated with a reduction in cardiac output [64]. However, a systematic review and meta-analysis involving 19 trials based on healthy or unhealthy adults revealed that although grape seed extract supplementation significantly reduced DBP (−2.20 mmHg, 95% confidence interval (CI): −3.79 to −0.60) and heart rate (−1.25 bpm, 95% CI: −2.32 to −0.19), it had no significant effects on flow-mediated dilation (1.02%, 95% CI: −0.62 to 2.66) and SBP (−3.55 mmHg, 95% CI: −7.59 to 0.49) [65]. These inconsistent results might be due to differences in dose and duration of grape seed extract intervention and the characteristics of the participants. 

Grape supplementation, in addition to other health-benefiting behaviors, could have a protective effect on the cardiovascular system. For example, a double-blind, placebo-controlled RCT revealed that daily intake of 400 mg grape seed proanthocyanidin extract for 12 weeks significantly decreased mean SBP by 13 mmHg in middle-aged participants with prehypertension, and ad hoc analysis revealed that grape seed proanthocyanidin treatment, along with non-smoking, not only decreased mean SBP but also decreased DBP and improved distensibility, stiffness parameter β, incremental elastic modulus and pulse wave velocity [66]. Moreover, compared with a calorie-restricted diet (about 250 kcal less than the estimated energy requirement), grape seed extract (300 mg/day) cotreatment with a calorie-restricted diet resulted in a more significant improvement in cardiovascular risk factors in obese or overweight adult individuals, such as by improving the blood lipid profile, visceral adiposity index and atherogenic index of plasma [67]. 

In general, grape can effectively prevent cardiovascular diseases by lowering blood pressure, reducing oxidative stress and improving cardiac remodeling. In addition, grape cointervention with other health-benefiting behaviors, such as not smoking or a calorie-restricted diet, could induce a cardioprotective effect.

### 3.6. Antidiabetic Activity

Diabetes mellitus is a chronic metabolic disease caused by absolutely or relatively insufficient insulin secretion and certain degrees of insulin resistance [68]. Controlling glycemia is a practical approach to the prevention and treatment of diabetes mellitus. Some natural products can lower the level of postprandial blood glucose by inhibiting the activities of amylase and α-glucosidase, which are important enzymes with respect to delaying the hydrolysis of complex sugars and decreasing the release of glucosyl units into the blood [46]. A study by Kong et al. revealed that grape seed aqueous extracts exerted a stronger inhibitory effect on the activities of α-amylase and α-glucosidase than acarbose [69]. Insulin is secreted by pancreatic β cells and is the only hormone with hypoglycemic ability in the body, which it exerts by accelerating glucose utilization and inhibiting glucose production. Low-quality and dysfunction of pancreas and Langerhans islets could cause insufficient secretion of insulin, promoting the development of diabetes mellitus. A study by Irak et al. revealed that grape seed extract prevented histopathological changes of pancreas and improved the function and structure of the pancreas and Langerhans islets in rat with streptozotocin-induced diabetes [9]. Insulin resistance is also an important pathogenic factor with respect to diabetes mellitus, which is caused by the low sensitivity of peripheral tissue to insulin. It was reported that virgin grape seed oil significantly alleviated insulin resistance in high-fat-diet-fed mice. The study results further suggest that polyphenols might be the most essential factor regulating insulin resistance [70]. 

Diabetes mellitus could lead to a series of complications, severely endangering health. Diabetic nephropathy and diabetic retinopathy are among the most common and concerning diabetic complications. Increasing evidence has revealed that grape seed extracts and proanthocyanidins are effective in the prevention and treatment of diabetic nephropathy and diabetic retinopathy. It was reported that grape seed proanthocyanidin extract prevented the development of diabetic nephropathy in streptozotocin-induced diabetic rats by reducing endoplasmic reticulum stress-induced apoptosis via the caspase-12 pathway [71]. Grape seed proanthocyanidin extract also reduced renal damage in rats with diabetes mellitus by ameliorating oxidative-stress-mediated injury by activating the Nrf2 signaling pathway [72]. In addition, grape seed extract protected against retinal injury in streptozotocin-induced diabetic rats by attenuating retinal Muller cell gliosis and alleviating oxidative stress by upregulating the expression of Nrf2-responsive antioxidant genes [73]. Moreover, grape seed proanthocyanidin extract inhibited the damage of photoreceptor cells in diabetic mice by protecting cells from hyperglycemia-induced degeneration and apoptosis through the inhibition of thioredoxin (Trx)/apoptosis signal-regulating kinase 1/the Trx-interacting protein signaling pathway [74].

In summary, grape has showed considerable anti-diabetic activity by inhibiting the activities of amylase and α-glucosidase, improving the function and structure of the pancreas and Langerhans islets and alleviating insulin resistance. Moreover, grape can prevent the development of diabetic complications, such as diabetic nephropathy and diabetic retinopathy.

### 3.7. Hepatoprotective Activity

Nonalcoholic fatty liver disease (NAFLD) is among the world’s most concerning chronic diseases. Some studies have shown that grape and its bioactive compounds, especially polyphenols, could play a role in the prevention and treatment of NAFLD. For instance, a study revealed that polymerized anthocyanin from grape skin extract decreased fat accumulation and steatosis in the liver, improved liver function and blood lipids and regulated lipid metabolism in high-fat-diet-induced NAFLD mice [75]. In another study, 10 mL of egg yolk and 1.5 g of pure cholesterol were intraorally administered to induce NAFLD in albino rabbits fed a regular diet, whereas grape leucoanthocyanidin intervention at a dose of 50 mg/kg for 100 days ameliorated histopathological changes in the liver and reduced hepatic steatosis in these rabbits [76].

Liver injury is also a severe public health problem. An increasing number of studies have reported that grape can protect the liver from hepatotoxicity induced by diverse chemicals, such as toxic organic substances, heavy metals and some drugs. Grape seed proanthocyanidins were reported to exert a protective effect against carbon-tetrachloride-induced acute liver injury in mice, which might be associated with the scavenging of free radicals, inhibition of lipid peroxidation and preservation of immune function [77]. Another study by Liu et al. showed that grape seed procyanidin extract increased cell viability, inhibited lactate dehydrogenase release and decreased ROS levels in primary hepatocytes treated with lead acetate in vitro [78]. Furthermore, grape significantly attenuated liver injury induced by lead in rats via the activation of the Nrf2 pathway in vivo [78]. Doxorubicin is an anthracycline commonly used in chemotherapy, although it could cause hepato-toxicity. In a study, doxorubicin-induced oxidative stress affected liver function and changed liver morphology in rats, whereas grape seed and skin extract effectively protected the liver from the toxicity, which suggests that grape extract could serve as a safe adjuvant for chemotherapy [79].

In summary, grape and its bioactive compounds were effective in the prevention and management of NAFLD and liver injury induced by a variety of chemicals, such as carbon tetrachloride, lead acetate and doxorubicin.

### 3.8. Anticancer Activity

Cancer is a global threat to the health and life of human beings. Grape and its bioactive compounds, such as proanthocyanidins, have shown anticancer activity against various cancers, such as liver, bladder, prostate and cervical cancers, and the underlying mechanisms have been extensively studied [3,68,80]. Grape possesses antiproliferative and cell-cycle-arrest-inducting capacities, which considerable contribute to its anticancer potential. For example, a study reported that a diet supplement with mixed powder of grape seed and grape skin prevented tumor development of 47% of mice inoculated with Ehrlich ascites carcinoma and markedly decreased the tumor volume and weight in mice with cancer by 93.9% and 86.3%, respectively, which was associated with the inhibition of cell proliferation and the induction of cell cycle arrest in the G1 phase, as well as apoptosis [10]. In addition, lipophilic grape seed proanthocyanidin effectively blocked the progression of the cell cycle in the G2/M phase and induced apoptosis in HeLa cell lines by increasing the level of ROS, in addition to significantly inhibiting the growth of an HeLa-derived xenograft tumor in zebrafish, displaying an anti-cervical cancer effect [81]. 

The induction of cancer cell apoptosis or autophagy plays an imperative role in the preventive and therapeutical effect of grape on cancers. Grape seed proanthocyanidin extract significantly induced apoptosis and inhibited the growth of hepatocellular carcinoma cells by inhibiting the activation of the MAPK/Akt pathway [82]. Another study showed that grape seed proanthocyanidins induced autophagy, increased apoptosis and reduced the expression of survivin in HepG2 cells and significantly inhibited the growth of cancer cells in HepG2-derived xenograft nude mice by inducing the phosphorylation of MAPK-pathway-associated proteins [83]. Moreover, grape suppressed angiogenesis to cut off the supply of nutrition and energy for cancer growth, thus preventing the development of cancer. It was found that grape powder inhibited angiogenesis, improved the prostate neoplastic phenotype, protected against hyperactive cell survival pathways (Akt and androgen receptor pathways), attenuated inflammation and reduced circulating levels of oncogenic microRNAs in *Pten*-deficient mice (the most commonly lost cancer suppressor gene in male patients diagnosed with prostate cancer) [84]. Additionally, grape could be effective in inhibiting the migration and invasion of cancers, which are distinctive characteristics of malignant tumors. For instance, grape seed proanthocyanidins were reported to markedly suppress the migration and invasion of T24 and 5637 bladder cancer cells by reversing epithelial–mesenchymal transition through the inhibition of the transforming growth factor-β (TGF-β) signaling pathway [85].

Grape is able to ameliorate multidrug resistance, which is a considerable obstacle and challenge associated with cancer treatment. For example, a study revealed that human leukemia cell line HL-60 and HL-ADR cells were resistant to a variety of chemotherapeutic drugs, including cytarabine, adriamycin, vincristine, daunorubicin, mitoxantrone, pirarubicin, homoharringtonine and etoposide, whereas some of agents, such as cytarabine and adriamycin in combination with grape seed proanthocyanidin extract reversed the multidrug resistance. This effect was associated with the inhibition of the PI3K/Akt signaling pathway, which led to the downregulated expression of multidrug-resistance-associated protein 1, multidrug resistance protein 1 and lung-resistance-related protein [86]. Oligomeric proanthocyanidins from grape seed extract were reported to sensitize both acquired chemoresistant colorectal cancer cells (HCT116-FOr cells) and innately chemoresistant colorectal cancer cells (H716 cells) to chemotherapeutic drugs oxaliplatin and 5-fluorouracil, and their coadministration with chemotherapeutic drugs significantly decreased chemoresistant xenograft tumor growth in mice. These effects were associated with the inhibition of adenosine triphosphate-binding cassette transporter proteins [87].

Together, grape and its bioactive components show promise with respect to the prevention and treatment of a variety of cancers, with related mechanisms including suppression of cancer cell proliferation, induction of cell cycle arrest, promotion of autophagy or apoptosis, reduction in angiogenesis, inhibition of migration and invasion and alleviation of multidrug resistance.

### 3.9. Other Health Benefits

**Figure 2:**
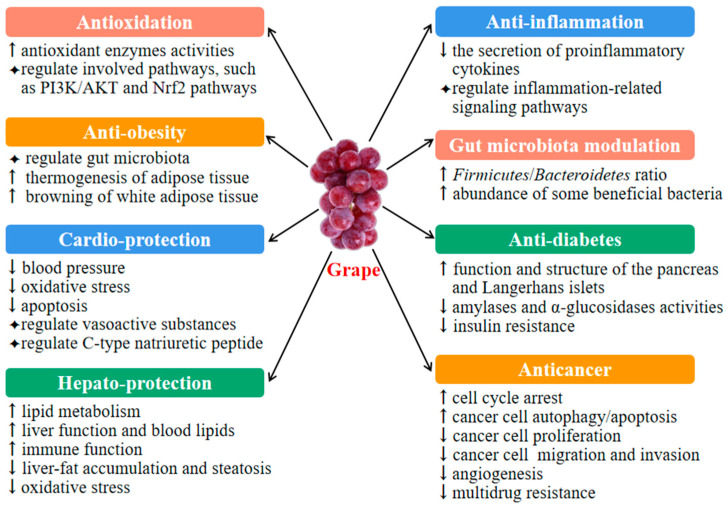
The health benefits of grape. Grape showed several bioactivities with different mechanisms of action. The up arrow ↑ represents increase, down arrow↓represents decrease, and the ◆ represents regulate.

Grape and its bioactive compounds also have other health benefits, such as neuroprotective and antiaging activities. A study by Ben Youssef et al. revealed that red grape seed and skin extract effectively protected dopamine neurons from 6-hydroxydopamine-induced toxicity by decreasing apoptosis, oxidative stress and inflammation and prevented neuronal loss and improved motor function in a mouse model of Parkinson’s disease [88]. In addition, 10-day treatment with 500 mg/kg bw grape seed procyanidins prevented certain aging processes in aged female rats, such as visceral adiposity, pancreas dysfunction and tumor development [89]. The health benefits and underlying mechanisms of grape and its bioactive compounds are showed in Table 2 and Figure 2.

## 4. Applications of Grape in the Food Industry

**Figure 3:**
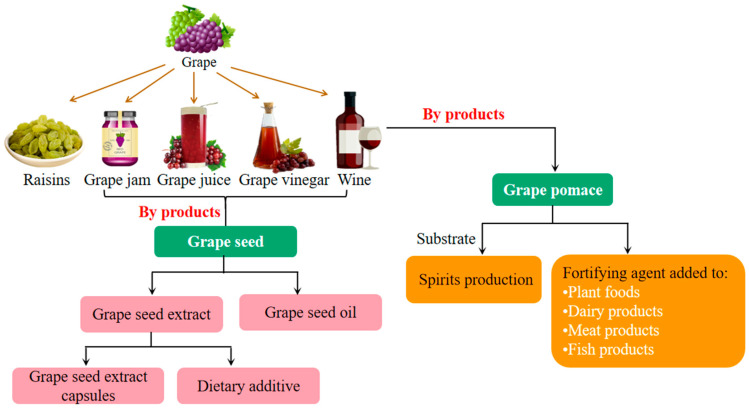
The applications of grape in the food industry.

An estimated 75 million tons of grape is produced annually worldwide. The vast majority of grapes are consumed as fresh fruit or used as food materials to produce wine, jam, juice and raisins (Figure 3), among which winemaking accounts for the largest proportion of grape use in the food industry [3]. In addition, the byproducts of grape, such as grape pomace and grape seed, have aroused increasing research interest due to their considerable potential application in the food industry [95].

### 4.1. Winemaking

Approximately 50% of grape is reportedly used for wine production, which is one of the most vital agro-industrial sectors worldwide. Wine is the second most consumed alcoholic beverage behind beer. According to data from the International Organization of Vine and Wine, worldwide wine consumption totaled 244 million hectoliters in 2019 [96,97]. Wine is produced by fermentation with fresh grape or grape must, which is carried out by yeasts via the consumption grape sugars, subsequently producing ethanol and CO_2_ [98]. A variety of other substances, such as organic acids, polysaccharides and polyphenols, are also produced or transported from grape in the processes of the wine production, storage and maturation, some of which are responsible for the sensory properties (such as flavor, color and taste) and health benefits of wine [99]. Many health benefits of wine are attributed to resveratrol, which is mainly found in grape skins and enters into wine through maceration and fermentation. As the “French paradox” reveals, populations that exhibit regular and moderate consumption of wine have lower incidences rate of coronary heart disease, despite the daily intake of high-fat diets. In addition, wine has considerable potential with respect to the prevention and management of other diseases, such as obesity, neurodegenerative diseases and cancers [100].

### 4.2. Application of Grape Pomace in the Food Industry

Considering the extensive worldwide winemaking industry, large amounts of grape pomace or wine pomace are produced, accounting for 15–30% of the initial weight of the grape and mainly consisting of grape peel and seed. The exploitation and utilization of grape pomace in the food industry could prevent environmental issues caused by the disposal of grape pomace, reducing he waste of resources and obtaining more economic and nutritional benefits [97]. A traditional application of grape pomace in the food industry is as a substrate for the production of some spirits by distillation. Recently, the exploitation and application of grape pomace in the production of fortified foods has gained increasing scientific and industrial interest, owing to its considerable promise. Many studies have revealed that the addition of grape pomace to a wide range of food products, such as plant, dairy and meat products, could improve the nutritional composition of the final products and increase their values [18,101]. For example, some plant foods (such as muffins, biscuits, bread, cookies, extruded cereals, noodles, pasta and pancakes) with grape pomace inclusion showed increased contents of dietary fiber and polyphenols, as well as improved antioxidant activity [101]. In addition, some studies showed that meat or fish products fortified with grape pomace could inhibit lipid oxidation and prolong storage or shelf life [102,103]. However, the fortification of grape pomace in high concentrations might adversely affect the textural and sensory properties of final products. In the future, more efforts are needed to diminish the unfavorable textural and sensory changes caused by the addition of grape pomace. Furthermore, additional in vitro, in vivo and clinical studies are needed to examine the health efficacy of such products.

### 4.3. Application of Grape Seed in the Food Industry

Grape seed is an easily obtained byproduct of grape juice and wine processing. Grape seed, storing the majority of polyphenolic content of grape, exhibits rich bioactivities, with promising application prospects in the food industry. Grape seed extract, a compound rich in polyphenolics, has been generally recognized as safe (GRAS) by the Food and Drug Administration and is commercially sold as a dietary additive listed on the “Everything Added to Food in the United States (EAFUS)” to improve the overall quality of products and extend their shelf life [21]. For example, the addition of grape seed extract to Western-style smoked sausage improved its color and extended its shelf life due to the strong antioxidant properties of grape seed extract [104]. At present, nitrite is widely used as an additive to prevent meat spoilage and manufacture meat products, potentially allowing them to be kept at ambient temperatures, whereas nitrite could react with secondary amines to generate harmful substances, such as N-nitramine, which could induce the occurrence of cancer. In particular, grape seed extract could significantly decrease residual nitrite and inhibit the formation of N-nitrosamine [104].

Considerable interest has been focused on the use of grape seed extracts as raw material to develop products with nutritional value and health benefits, with considerable popularization and application prospects. For example, grape seed extract capsules have been developed and sold by some companies as dietary nutritional supplement products to protect humans from oxidative damage and to maintain health. Moreover, due to its solubility in water and ethanol, grape seed extract has considerable potential as a beverage added to produce products with health benefits that could satisfy the taste standards of the public [105,106].

Grape seed is also a valuable source of oils. Grape seed oil is rich in unsaturated fatty acids (especially linoleic acid) and lipid-soluble vitamins (such as vitamins A, D and E), conferring grape seeds oil with various health-promoting activities [22]. Recently, some lipid-embedded substances in grape seed oil, such as tocopherols, phytosterols and phenols, have attracted increasing attention from researchers because of their diverse biological activities, such as antioxidant and anti-inflammatory activities [107]. In this respect, the recovery of oil from grape seeds is of considerable significance with respect to the exploitation and utilization of grape. Some grape seed oil products have been sold by companies and are consumed as part of the daily diet.

## 5. Conclusions

Grape is one of the most produced and consumed fruits in the world, representing a source of polyphenols, especially proanthocyanidins, anthocyanins and resveratrol. The various polyphenols considerable contribute to the diverse biological activities of grape, such as antioxidant, anti-inflammatory, antidiabetic, anticancer and cardioprotective activities. As a popular and nutritious fruit, grape has been widely used as a raw material to produce a variety of products, such as wine, grape jam, grape juice and raisins. The majority of grapes are used to produce wine, which is associated with a variety of health benefits for humans. In addition, the application of grape byproducts, such as grape pomace and grape seed, has been extensively studied, with progress reported in the food industry. Some food products derived from these byproducts have been developed, such as grape seed extract capsules and grape seed oil. In the future, more bioactive compounds in grape should be isolated and identified. Their bioactivities should be evaluated, and their underlying mechanisms of action should be explored. In addition, the applications of grape pomace and grape seed extract should be studied in more food. Furthermore, more clinical trials should be carried out to confirm the health benefits of grape and its byproducts in human beings.

## Figures and Tables

**Table 1:** The 78 phenolic compounds in grape pulp samples [26].

Compound Name	Compound Name	Compound Name	Compound Name
Gallic acid 4-O-glucoside	Caffeic acid	Hesperetin 3′-sulfate	Quercetin 3-O-arabinoside
Ellagic acid arabinoside	3-Caffeoylquinic acid	Neoeriocitrin	Violanone
Gallic acid	3-p-Coumaroylquinic acid	Hesperidin	2′-Hydroxyformononetin
Protocatechuic acid 4-O-glucoside	3-Feruloylquinic acid	Hesperetin 3′-O-glucuronide	5,6,7,3′,4′-Pentahydroxyisoflavone
4-Hydroxybenzoic acid 4-O-glucoside	Ferulic acid	Rhoifolin	3′-Hydroxygenistein
Schisandrin C	1,5-Dicaffeoylquinic acid	Cirsilineol	6”-O-Acetylglycitin
2-Hydroxybenzoic acid	3-Sinapoylquinic acid	Cirsilineol	Arbutin
Paeoniflorin	3,4-Dihydroxyphenylacetic acid	Diosmin	Isopimpinellin
3-O-Methylgallic acid	2-Hydroxy-2-phenylacetic acid	Chrysoeriol 7-O-glucoside	4-Hydroxybenzaldehyde
Cinnamic acid	Dihydroferulic acid 4-O-glucuronide	Myricetin 3-O-arabinoside	p-Anisaldehyde
Caffeoyl tartaric acid	Dihydrocaffeic acid 3-O-glucuronide	Procyanidin dimer B1	p-Anisaldehyde
p-Coumaric acid 4-O-glucoside	Myricetin 3-O-rhamnoside	Kaempferol 3-O-xylosyl-glucoside	4″-O-Methylepigallocatechin 3-Ogallate
m-Coumaric acid	3-Hydroxyphloretin 2′-O-glucoside	Myricetin 3-O-rutinoside	Carnosic acid
Ferulic acid 4-O-glucoside	Phloridzin	Spinacetin 3-O-(2	Hydroxytyrosol 4-O-glucoside
Cyanidin 3-O-(2-O-(6-O-(E)-caffeoyl-D glucoside)-D-glucoside)-5-O-Dglucoside	Kaempferol 3-O-(2″-rhamnosylgalactoside) 7-O-rhamnoside	Patuletin 3-O-glucosyl-(1->6)-[apiosyl (1->2)]-glucoside	3′,4′,7-Trihydroxyisoflavanone
Hydroxycaffeic acid	Dihydromyricetin 3-O-rhamnoside	Myricetin 3-O-galactoside	7-Oxomatairesinol
Caffeoyl glucose	Procyanidin trimer C1	Kaempferol 3,7-O-diglucoside	Pinoresinol
Ferulic acid 4-O-glucuronide	Caffeic acid 3-O-glucuronide	Quercetin 3′-O-glucuronide	2,3-Dihydroxybenzoic acid
Caffeic acid 4-sulfate	(+)-Catechin	Kaempferol 3-O-glucosyl-rhamnosylgalactoside	Resveratrol 5-O-glucoside
Feruloyl tartaric acid	(+)-Gallocatechin		

**Table 2:** Health benefits of grape.

Study Type	Model	Product (Component)	Treatment	Main Effects and Related Mechanisms	Ref.
***Antioxidant ability***					
In vitro	/	Grape extract (hydroxycinnamic acids, flavan-3-ols and tannins)	/	Exhibited antioxidant power, including ORAC, DPPH and ABTS radical-scavenging activities; FRAP, CUPRAC	[41]
In vitro	/	Grape pulp, peel and seed extracts (gallic acid, quercetin, catechin, chlorogenic acid, caffeic acid and p-coumaric acid)	/	Antioxidant capacity (determined by DPPH radical-scavenging activity and FRAP and TEAC assays): grape seed > grape peel > grape pulp	[90]
In vitro	/	Pulp, peel and seed extracts (epicatechin, catechin gallate, gallic acid, rutin and resveratrol)	/	Antioxidant capacity (determined by FRAP and TEAC assays): grape seed > grape peel > grape pulp; these results are in accordance with the respective TPC	[4,19]
In vitro	LPS-induced oxidation in human Caco-2 colon cells	Grape seed extract	Culture: 12.5 μg/mL for 24 h	Reduced the generation of intracellular ROS and mitochondrial superoxide and increased the gene expression of antioxidant enzymes (GSR, SOD and GP_X_)	[42]
In vitro	H_2_O_2_-induced oxidation in PC12 cells	Grape seed proanthocyanidins	Culture: 5, 10 or 25 μM for 24 h	Protected against oxidative damage via the PI3K/Akt signaling pathway	[44]
In vivo	TNBS-treated Wistar rats	Grape peel powder	Diet supplementation: 8% for 15 d	Ameliorated TNBS-induced oxidative damage by improving the activities of antioxidant enzymes (SOD and CAT) and decreasing oxidation and NO levels	[43]
In vivo	A varicocele model in Wistar rats	Grape seed proanthocyanidin extract	Oral administration: 250 mg/kg for 4 weeks	Reduced varicocele-induced testicular oxidative damage by activating the Nrf2 pathway	[45]
***Anti-inflammatory activity***					
In vitro	TNF-α-treated gastric epithelial cells	Grape extracts (phenolic compounds)	Culture: 5–100 μg/mL for 6 h	Inhibited TNF-α-induced IL-8 release	[2]
In vivo	Transgenic mice overexpressing TNF	Whole grape powder	Diet supplementation: 5% or 10% for 4 weeks	Suppressed human TNF-mediated inflammation and improved the symptoms of inflammatory arthritis	[47]
In vivo	TNBS-treated Wistar rats	Grape peel powder	Diet supplementation: 8% 15 d before and 7 d after TNBS treatment	Ameliorated inflammation by downregulating the NF-κB pathway	[43]
In vivo	DSS-induced inflammation in hybrid piglets	Grape seed meal (catechins, epicatechins and procyanidins)	Diet supplementation: 8% for 30 d	Counteracted the inflammatory response by decreasing the production of proinflammatory mediators, inhibiting the MAPK and NF-κB pathways	[48]
In vivo	DSS-induced inflammation in C57BL/6 mice	Grape seed polyphenol extract	Oral administration: 500 and 750 mg/kg for 6 d	Decreased inflammatory infiltration by inhibiting the mRNA expression of inflammatory cytokines (IL-1β, IL-6 and TNF-α) and reducing the phosphorylation of STAT3	[91]
In vivo	Cigarette-smoke-exposed SD rats	Grape seed proanthocyanidin	Intratracheal injection: 30 mg, 2 mL/kg for 6 months	Inhibited inflammation through the PPAR-γ/COX-2 pathway	[49]
***Gut microbiota modulatory ability***					
In vivo	HFFD-fed C57BL/6 J mice	Grape extract	Drinking: 1% *w*/*w* dissolved in water	Restored the disturbance of gut microbiota by improving the ratio of *Firmicutes*/*Bacteroidetes* and the abundance of the *Bifidobacteria*, *Clostridia* and *Akkermansia* genera	[6]
In vivo	PhIP-treated Wistar rats	Grape seed extract	Intragastric administration: 60 mg/kg/d for 6 weeks	Maintained the homeostasis of gut microbiota, especially by preventing a decrease in *Lactobacillus* abundance induced by PhIP, thereby ameliorating colonic injury	[51]
In vivo	HFD-fed C57BL/6 J mice	Grape seed polyphenol extract	Oral administration: 200 mg/kg/d for 7 d	Promoted the recovery of gut microbiota after treatment with an antibiotic cocktail	[52]
***Antiobesity activity***					
In vivo	HFFD-fed C57BL/6 Cnc mice	Grape extract	Drinking: 1% *w*/*w* dissolved in water	Prevented obesity by restoring the dysbiosis of gut microbiota, subsequently modulating serum bile acid and promoting GPBAR1 in BAT, thereby activating thermogenesis of BAT	[6]
In vivo	Western-diet-fed C57BL/6 J mice	Grape polyphenol extract (B-type proanthocyanidins)	Diet supplementation: 1% for 23 weeks	Improved body composition and reduced adiposity, which were associated with a reduced level of gut butyrate and an increased abundance of *Akkermansia muciniphila*	[92]
In vivo	HFD-fed C57BL/6 J mice	Grape seed flour	Diet supplementation: 2.5% or 7.5% for 17 weeks	Reduced body weight gain and improved lipid profiles in hepatic and serum by increasing thermogenesis of BAT and energy expenditure	[55]
In vivo	HFD-fed C57BL/6 J mice	Grape seed proanthocyanidin extract	Oral administration: 200 mg/kg/d for 8 weeks	Reduced overweight by increasing the thermogenesis of adipose tissue, enhancing the browning of white adipose tissue and modulating gut microbiota	[56]
In vivo	Cafeteria-diet-treated aged rats	Grape seed proanthocyanidin extract	Intragastric administration: 500 mg/kg for a 10-day preventive treatment or an 11-week simultaneous treatment	Simultaneous treatment effectively reduced body weight, total adiposity and liver steatosis, whereas preventive treatment only decreased mesenteric adiposity	[57]
Double-blind RCT	40 obese or overweight subjects	Grape seed extract	Capsules: 300 mg grape seed extract daily for 12 weeks	Reduced body weight, BMI, waist circumference and waist-to-hip ratio	[58]
***Cardioprotective activity***					
In vitro and in vivo	EA.hy926 endothelial cells; DOCA-salt-treated SD rats	Grape extract	Culture: 0–100 μg/mL; diet supplementation: 0.03% (*w*/*w*) for 3 weeks; oral administration: 500 mg/kg for 5 d	Increased the production of NO and restored endothelial dysfunction and hypertension induced by DOCA salt by activating endothelial NO synthase and the PI3K/Akt pathway	[61]
In vitro and in vivo	MCEC-1 cells; mouse model of myocardial infarction	Tuscany Sangiovese pure grape juice	Cell culture: 10–100% *v*/*v*; Diet supplementation for mice: 25% *v*/*v*, 200 µL/d for 4 weeks	Protected the myocardium from an ischemic microenvironment and showed a protective effect on infarcted hearts by regulating gene expression of CNP	[7]
In vitro and in vivo	H9C2 cells; C57BL/6J mice with myocardial infarction	Grape seed proanthocyanidin extract	Culture: 40 μg/mL; Intragastric administration: 200 mg/kg/d for 14 days;	Improved cardiac remodeling and dysfunction induced by myocardial infarction and prevented apoptosis of cardiomyocytes under hypoxic conditions via the PI3K/Akt pathway	[63]
Randomized, double-blind, crossover test	Normal-body-weight and obese male subjects	Grape seed extract	300 mg in 2 capsules per administration twice, 1 week apart	Lowered SBP and MAP in both NBW and obese males subjects; the efficacy in obese male patients might be related to a reduction in cardiac output	[64]
Double-blind, placebo-controlled RCT	Middle-aged participants with prehypertension	Grape seed proanthocyanidin extract	200 or 400 mg in tablets daily for 12 weeks	400 mg/d: lowered mean SBP by 13 mmHg; 400 mg/d and non-smoking: significantly lowered mean SBP and DBP and improved PWV, distensibility, incremental elastic modulus and stiffness parameter β	[66]
Double-blind, placebo-controlled RCT	Obese or overweight adults	Grape seed extract	300 mg/d for 12 weeks	Along with a calorie-restricted diet, improved cardiovascular risk factors, such as visceral adiposity index, blood lipid profile and plasma atherogenic index	[67]
***Antidiabetic activity***					
In vitro	Amylases and α-glucosidases	Grape seed aqueous extracts (catechin and epicatechin)	Culture: 25.25 or 66.68 μg/mL	Exerted a stronger effect than acarbose in inhibiting the activities of amylases and α-glucosidases	[69]
In vivo	STZ-induced diabetic rats	Grape seed extract	Intragastrical administration: 0.6 mL/rat for 20 d	Improved the functions and structures of pancreas and Langerhans islets and enhanced enzyme activities	[9]
In vivo	STZ-induced diabetic rats	Grape seed extract	Gavage: 400 mg/kg/d for 28 d	Increased pancreatic mass	[93]
In vivo	HFD-fed mice	Virgin grape seed oil (polyphenols)	Diet supplementation: 29% *w*/*w* total oil for 15 weeks	Reduced blood glucose and alleviated insulin resistance	[70]
In vivo	HFD-fed mice	Grape seed flour	Diet supplementation: 10% for 5 weeks	Lowered fasting glucose concentration and alleviated insulin resistance	[94]
In vivo	Diabetic rats	Grape seed proanthocyanidin extract	Intragastric administration: 250 mg/kg/d for 16 weeks	Prevented renal damage by lowering endoplasmic reticulum stress-mediated apoptosis via the caspase-12 pathway	[71]
In vivo	Diabetic rats	Grape seed proanthocyanidin extract	Intragastric administration: 125 or 250 mg/kg/d for 8 weeks	Lowered blood glucose and reduced renal injury by inhibiting oxidative stress-mediated damage by activating the Nrf2 signaling pathway	[72]
In vivo	Diabetic rats	Grape seed extract	Intragastric administration: 250 mg/kg/d for 16 weeks	Lowered blood glucose and prevented retinal injury by decreasing retinal Muller cell gliosis and oxidative stress by activating the Nrf2 signaling pathway	[73].
In vitro and in vivo	Macroglial Muller cells; diabetic mice	Grape seed proanthocyanidin extract	Culture: 10 or 20 μg/mL for 72 h; Oral gavage: 200 or 300 mg/kg/d for 10 weeks	Inhibited photoreceptor cell damage by protecting them from hyperglycemia-induced degeneration and apoptosis via the inhibition of the Trx/ASK 1/Txnip signaling pathway	[74]
***Hepatoprotective ability***					
In vivo	HFD-induced NAFLD mice	Polymerized anthocyanin from grape skin extract	Oral gavage: 400 mg/kg/d for 4 weeks	Decreased hepatic fat accumulation and steatosis, improved liver function and blood lipids and regulated lipid metabolism	[75]
In vivo	Albino rabbits additionally administered 10 mL egg yolk and 1.5 g pure cholesterol	Grape leucoanthocyanidin	Oral gavage: 50 mg/kg/d for 100 d	Recovered hepatic tissue and reduced hepatic steatosis, which is reflected a reduced NAFLD activity score from 6 to 4	[76]
In vivo	CCl_4_-treated Kunming mice	Grape seed proanthocyanidins	Oral gavage: 50, 250 or 500 mg/kg for 10 d	Protected liver from acute injury by scavenging free radicals, inhibiting lipid peroxidation, preserving immune function and improving antioxidant capacity	[77]
In vitro and in vivo	Lead-acetate-treated primary hepatocytes and rats	Grape seed procyanidin extract	Culture: 100 μg/mL for 2 h; Oral gavage: 200 mg/kg/d for 8 weeks	Increased cell viability, inhibited LDH release and decreased ROS levels in primary hepatocytes and alleviated liver injury in rats by activating the Nrf2 pathway	[78]
In vivo	Doxorubicin-treated rats	Grape seed and skin extract (polyphenols)	Oral gavage: 500 mg/kg/d for 8 d	Prevented hepatotoxicity induced by doxorubicin	[79]
***Anticancer activity***					
In vivo	Mice inoculated with Ehrlich ascites carcinoma	Mixed powder of grape seeds and grape skin	Diet supplementation: 10% (*w*/*w*) 14 d before inoculation and continued throughout the experiment	Prevented 47% of tumor development and reduced volume and weight of tumors by 93.9% and 86.3%, respectively, by inhibiting cell proliferation, inducing cell cycle arrest in the G1 phase and promoting apoptosis	[10]
In vitro and in vivo	HeLa cell lines; HeLa-derived xenograft tumors in zebrafish	Lipophilic grape seed proanthocyanidin	Cell culture: 25–200 µg/mL for 24 and 48 h; Fish water: 4 and 8 μg/mL for 48 h	Induced cell cycle arrest in the G2/M phase, promoted apoptosis in vitro by increasing ROS levels and inhibited cancer growth in zebrafish	[81]
In vitro	Hepatocellular carcinoma cells	Grape seed proanthocyanidin extract	Culture: 12.5, 25, 50 and 100 µg/mL for 24 or 48 h	Induced apoptosis and inhibited cancer cell growth by inhibiting the MAPK/Akt pathway	[82]
In vitro and in vivo	HepG2 cells; HepG2-derived mouse xenograft model	Grape seed proanthocyanidins	Culture: 10 mg/L for 24 h; Oral gavage: 100 and 200 mg/kg	Induced autophagy/apoptosis, reduced survivin expression and inhibited cancer cell growth via the MAPK pathway	[83]
In vivo	*Pten*-deficient mice	Grape powder	Diet supplementation: 10% for 33 weeks	Reduced angiogenesis, attenuated inflammation, improved the prostate neoplastic phenotype, inhibited hyperactive cell survival via the Akt and AR pathways and reduced circulating levels of oncogenic microRNAs	[84]
In vitro	T24 and 5637 bladder cancer cells	Grape seed proanthocyanidins	Culture: 0–200 μg/mL for 24, 48 or 72 h	Suppressed migration and invasion by reversing EMT via inhibition of the TGF-β pathway	[85]
In vitro	Human leukemia cell line HL-60 and HL-ADR cells	Grape seed proanthocyanidin extract	Culture: 25 μg/mL for 24 h	Reversed the MDR of cancer cells to some drugs by inhibiting the PI3K/Akt pathway and down-regulating the expression of MDR-associated protein 1, MDR protein 1 and lung resistance-related protein	[86]
In vitro and in vivo	Acquired and innately chemo-resistant colorectal cancer cells (HCT116-FOr cells and H716 cells); HCT116-FOr xenograft mice	Oligomeric proanthocyanidins from grape seed extract	Culture: 100 ng/µL for 48 h; Oral gavage: 100 mg/kg for 6 weeks	Sensitized cancer cells to oxaliplatin and 5-fluorouracil (chemotherapeutic drugs) and decreased chemoresistant xenograft tumor growth in mice via the inhibition of ABC transporter proteins	[87]
***Other health benefits***					
In vitro and in vivo	Dopamine neurons; Parkinson′s disease mouse model	Red grape seed and skin extract	Culture: 500 and 1000 μg/mL; Intraperitoneal injection: 250 mg/kg in 10% ethanol	Protected dopamine neurons from 6-OHDA-induced toxicity by reducing apoptosis, oxidative stress and inflammation; prevented neuronal loss; and improved motor function in a Parkinson’s disease mouse model	[88]
In vivo	Aged female rats	Grape seed procyanidin extract	Diet supply: 500 mg/kg for 10 d	Prevented certain aging processes, such as visceral adiposity, pancreas dysfunction and age-related tumor development	[89]

Abbreviations: ABC, adenosine triphosphate-binding cassette; ABTS, 2,2′-azino-bis (3-ethylbenzothiazoline-6-sulfonic acid); Akt, serine-threonine kinase; AR, androgen receptor; ASK, apoptosis signal-regulating kinase; BAT, brown adipose tissue; BMI, body mass index; CAT, catalase; CCl_4_, carbon tetrachloride; CNP, C-type natriuretic peptide; COX-2, cyclooxygenase 2; CUPRAC, cupric-reducing antioxidant capacity; DBP, diastolic blood pressure; DOCA, deoxycorticosterone acetate; DPPH, 2,2-diphenyl-1-picrylhydrazyl; DSS, dextran sulfate sodium; EMT, epithelial–mesenchymal transition; FRAP, ferric-reducing antioxidant power; GPBAR1, G protein-coupled bile acid receptor 1; GP_X_, glutathione peroxidase; GSR, glutathione-disulfide reductase; HFD, high-fat diet; HFFD, high-fat and high-fructose diet; IL, interleukin; LDH, lactate dehydrogenase; LPS, lipopolysaccharide; MAP, mean arterial pressure; MAPK, mitogen-activated protein kinase; MDR, multidrug resistance; NBW, normal body weight; NAFLD, nonalcoholic fatty liver disease; NF-κB, nuclear factor kappa B; NO, nitric oxide; Nrf2, nuclear factor (erythroid-derived 2)-like 2; ORAC, oxygen radical absorbance capacity; PhIP, 2-amino-1-methyl-6-phenylimidazo (4,5-b) pyridine; PI3K, phosphoinositide-3 kinase; PPAR-γ, peroxisome proliferator-activated receptor γ; PWV, pulse wave velocity; RCT, randomized clinical trial; ROS, reactive oxygen species; SBP, systolic blood pressure; SOD, superoxide dismutase; SD, Sprague–Dawley; STAT3, signal transducer and activator of transcription 3; STZ, streptozotocin; TEAC, Trolox equivalent antioxidant capacity; TGF-β, transforming growth factor-β; TNBS, 2,4,6-trinitrobenzene sulfonic acid; TNF-α, tumor necrosis factor α; TPC, total phenolic content; Trx, thioredoxin; Txnip, Trx-interacting protein; 6-OHDA, 6-hydroxydopamine.

## Data Availability

All data presented within the article is available upon reasonable request from the corresponding author.
